# Specific Language Impairment and Executive Functions in School-Age Children: A Systematic Review

**DOI:** 10.7759/cureus.43163

**Published:** 2023-08-08

**Authors:** Rosa Alva Flores Camas, Jose E Leon-Rojas

**Affiliations:** 1 Postgraduate Department, Universidad Nacional de Chimborazo, Riobamba, ECU; 2 Department of Medicine, Universidad de las Américas, Quito, ECU; 3 Health Sciences Research Department, Medignosis, Quito, ECU; 4 Medical Research Department, NeurALL Research Group, Quito, ECU

**Keywords:** school-age children, language research, executive function deficits, executive function, specific language impairment

## Abstract

This systematic review examines the relationship between specific language impairment (SLI) and executive functions (EFs) in school-age children. The study investigates the impact of SLI on EF in comparison to children who develop normally. A total of 2,658 articles from three databases (PubMed, Scopus, and Virtual Health Library) were assessed for inclusion. Ultimately, 22 articles were selected for analysis, which contained information on both SLI and EF. The findings indicate that children diagnosed with SLI exhibit deficits, low performance, and, in some cases, significant deterioration in the development of EF when compared to typically developing children in 62%-91% of cases as early as three to four years of age; the most commonly found alterations were in working memory (including phonological, auditory, and visual/verbal memory), followed by deficits in attention, processing speed, inhibition, planning, cognitive flexibility, and internalized speech. We also discuss the close relationship and importance between language and EF in SLI children.

## Introduction and background

Specific language impairment (SLI) is a developmental disorder that is multifactorial in nature and has a high prevalence rate; it is known to be a risk factor for psychosocial and academic challenges that have a significant impact on school-age children when compared to their typically developing peers [1-3]. This study aims to elucidate the impact of SLI on executive functions (EFs) in children of school age, given the dearth of knowledge in this area.

Currently, there are two theoretical explanatory frameworks for SLI. The first posits that SLI arises from a particular grammatical deficit, which does not impact nonverbal abilities. The second suggests that SLI results from a broader processing impairment, stemming from the atypical development of the brain and structures that support the procedural memory system [2,3]. This impairment manifests as abnormalities in the memory process, leading to grammatical deficits and nonverbal disturbances that extend beyond language skills to include areas such as mathematics, motor skills, and attention [2,3]. Furthermore, it has been suggested that SLI is linked to executive dysfunction, encompassing deficits in inhibition, fluency, updating, and working memory. Despite ongoing research, the multifaceted construct of EF remains incompletely understood; however, there is consensus that EFs are advanced cognitive processes, comprising a diverse range of functions and procedures that facilitate purposeful behavior and self-management [4].

EFs undergo significant development from preschool age through adolescence. The primary components of EFs include cognitive flexibility, inhibition, and working memory, which are responsible for coordinating high-level EFs such as problem-solving strategies, planning, and reasoning [4,5]. Consequently, EFs play a critical role in the development of language disorders, including SLI [4,6]. Children with developmental disorders and those with SLI often exhibit executive deficits, as language plays a crucial role in cognitive self-regulation [4]. In the process of development, the practice of utilizing externally vocalized speech, also known as egocentric speech, to regulate one's thinking and behavior in early and middle childhood undergoes a transformation whereby it becomes internalized as a form of self-directed speech, known as internal speech [5,6]. This internal speech subsequently serves as a tool for development. Due to the strong correlation between language and executive functioning, there has been a surge of scholarly attention toward executive functioning in children with SLI. It has been suggested that children with SLI exhibit ineffective inner speech, resulting in suboptimal language utilization in certain non-linguistic activities [2,3].

The aim of this study is to consolidate the current scientific knowledge on the relationship between EFs and SLI in children of school age. Additionally, the quality of the studies and methodologies employed in the investigations pertaining to this disorder will be evaluated.

## Review

Methodology

This systematic review adhered to the guidelines set forth by the Preferred Reporting Items for Systematic Reviews and Meta-Analysis (PRISMA) [7].

Eligibility Criteria

Inclusion criteria for the articles were limited to those that presented a comprehensive analysis of EFs among school-aged children diagnosed with SLI; articles including healthy controls were also considered. Case series, cross-sectional, cohort, and case-control studies were considered for inclusion. The exclusion criteria comprised studies that involved patients with SLI and other concomitant pathology that might have an influence on EF, studies that did not analyze the type or frequency of EF defects, and studies that analyzed other aspects of EF such as the usefulness of diagnostic tools or treatments; case reports, literature reviews, systematic reviews, meta-analysis, letters to the editor, and conference abstracts were also excluded.

Language

The articles included in the study were limited to those written in English and Spanish.

Information Sources

The search strategy employed medical subheadings (MeSH) and text words pertaining to SLI, school-age children, and EFs. The databases searched in this study included Medline (PubMed), Scopus, and BVS (Virtual Health Library) from inception until January 25, 2023. 

Search Strategy

The database search parameters did not incorporate any restrictions. A systematic search was conducted using electronic databases including Scopus (Inception to January 25, 2023), Medline (Inception up to January 25, 2023), and BVS (Inception up to January 25, 2023). The search protocols and specific keywords used are provided in the supplementary files. The authors conducted an independent search and screening of the articles; in cases of disagreement regarding conflicting articles, discussion happened until a consensus was reached. 

Data Management

The articles that resulted from database exploration were imported into the web-based software Ryyan in order to mitigate data entry errors and minimize bias through the process of deduplicating references [8].

Selection Process

The aforementioned inclusion criteria were utilized by the authors to screen all titles and abstracts. Following this, literature that satisfied the inclusion criteria, even if the results were uncertain, underwent a thorough full-text review. Ryyan was used, independently and in a blinded fashion, by the two reviewers during the complete selection process and also during conflict resolution.

Data Items

The data extracted from the selected articles was compiled and organized in a Microsoft Excel (Microsoft Corporation, Redmond, USA) spreadsheet. The citation information of the article, including the author, publication year, country, and design, was included. Information regarding the total number of participants, sex, age, EF assessment, instrument or test used for assessment, and main results were extracted. Data was synthesized using proportions given the high degree of heterogeneity among the participants and the different testing used to evaluate EF.

Bias Assessment

In order to evaluate the potential for bias in the studies, the National Heart, Lung, and Blood Institute’s (NHLBI) study quality assessment tools were utilized. These tools comprised a set of questionnaires tailored to the specific type of study design being evaluated, including case-control studies, controlled intervention studies, cross-sectional, and observational cohort studies. The level of bias was categorized as low, moderate, or high based on the percentage of affirmative responses to the questions posed. A low risk of bias was indicated if 80% or more of the questions were answered affirmatively, while a moderate risk of bias was indicated if the percentage of affirmative responses was between 50% and 79%; a high risk of bias was assigned if less than 50% of the questions were answered affirmatively.

Results

**Figure 1:**
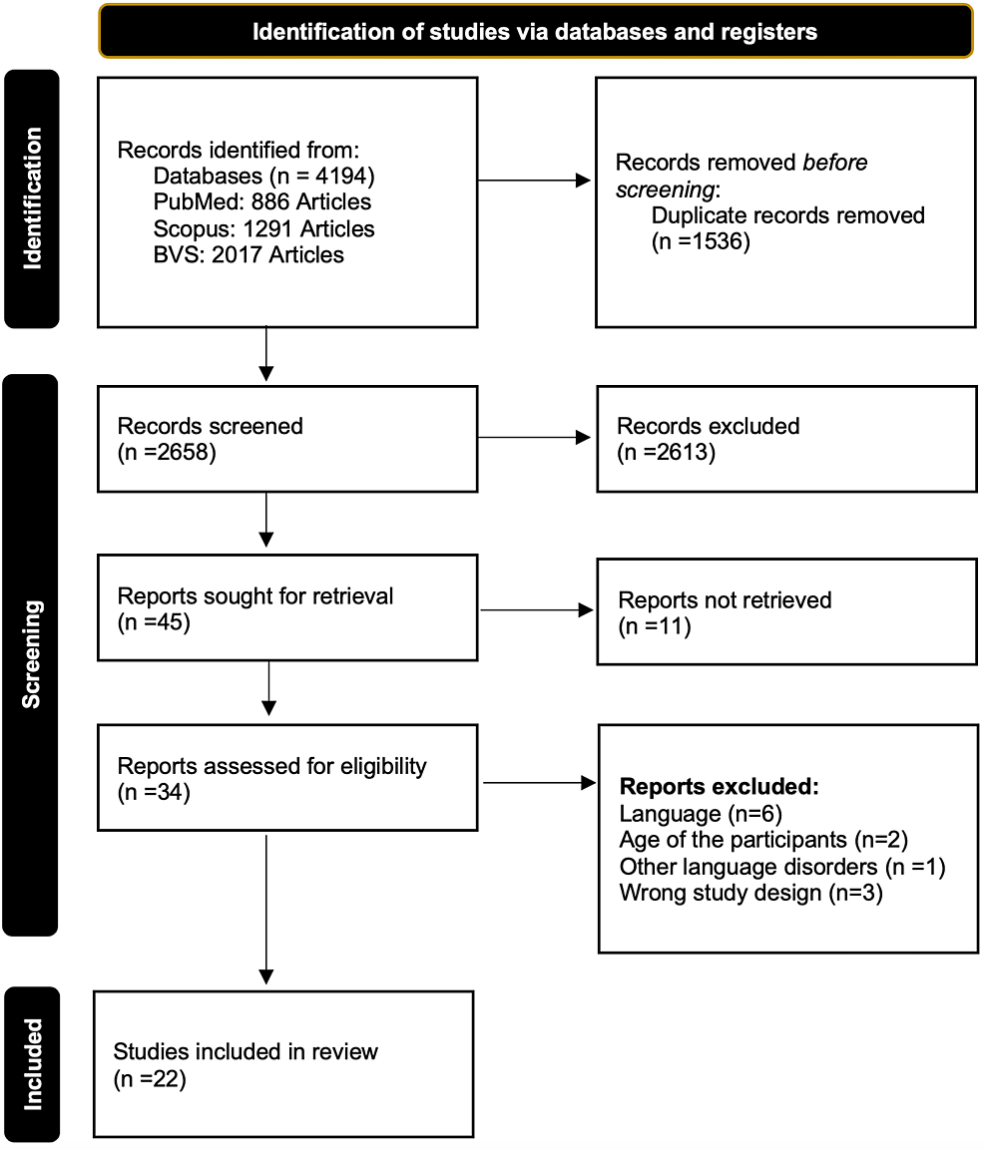
PRISMA Flowchart

The present study involved a comprehensive search and analysis of scientific articles from three different scientific platforms, resulting in a total of 2,658 articles assessed for inclusion by two independent and blinded reviewers. The selection process and its results can be seen in Figure 1; a total of 22 articles, which included 1,753 school-age children (952 children with SLI and 801 controls) ranging from 3 to 11 years of age were selected for this review [9-30]. The bias assessment we conducted on each study is available in Table 1; overall, 57% of the studies had a minimally low risk of bias and 43% had a moderately low risk of bias. The main results of the included studies can be seen in Table 2.

**Table 1:** Bias Assessment of Included Studies Bias assessment was performed using the study quality assessment tools. NHLBI, National Heart, Lung, and Blood Institute

Author (Year)	Study Design	Level of Bias
Katsos (2011) [9]	Cross-Sectional	Moderate
Guiraud (2017) [10]	Cross-Sectional	Moderate
Cabell (2011) [11]	Cross-Sectional	Low
Aziz (2015) [12]	Clinical Trial	Moderate
Ambiado-Lilo (2023) [13]	Cross-Sectional	Moderate
van Daal 2009 [14]	Cross-Sectional	Moderate
Pijnacker (2016) [15]	Cross-Sectional	Moderate
Lidstone (2012) [16]	Cross-Sectional	Low
Coelho (2013) [17]	Cross-Sectional	Low
Lukács (2015) [18]	Cross-Sectional	Low
Willinger (2017) [19]	Cross-Sectional	Low
Reichenbach (2016) [20]	Cross-Sectional	Low
Kapa (2019) [21]	Cross-Sectional	Moderate
Marton (2014) [22]	Cross-Sectional	Moderate
Quintero (2013) [23]	Cross-Sectional	Low
Im-Bolter (2006) [24]	Cross-Sectional	Low
Schuchardt (2013) [25]	Cross-Sectional	Low
Acosta (2015) [26]	Cross-Sectional	Low
Roello (2014) [27]	Cross-Sectional	Moderate
Vugs (2015) [28]	Cross-Sectional	Low
Kuusisto (2016) [29]	Cross-Sectional	Moderate
Henry (2012) [30]	Cross-Sectional	Low

**Table 2:** Main Results and Assessment Instruments of Each Included Study SLI, specific language impairment; TD, typical development; EF, executive function

Author (Year)	SLI Children (N)	TD Children (N)	Age Range (Years)	Assessment Instruments	Main Results
Roello (2014) [27]	60	58	4-6	Rustioni Language Comprehension, Peabody Receptive Communication Test (Peabody Picture Vocabulary Test III), Wechsler Intelligence Scale for Children, Flexible Item Selection Task (FIST), Try Day Night Similar to Stroop Tower of London Test	There is a clear deterioration of EF in SLI pre-school children, with 84% having poor performance on the tests in comparison with their TD peers.
Aziz (2015) [12]	87	80	4-10	Wechsler Nonverbal Ability Scale, Tower of London, Communication Checklist for Children 2nd Edition	Speech training improved self-regulatory speech and cognitive results in 93% of the children with SLI.
Reichenbach (2016) [20]	30	30	3-7	Colored Progressive Matrices, Snijders-Oomen Nonverbal Intelligence Test, Primary Intelligence Scale III (WPPSI III Receptive and Expressive Vocabulary Scores), Kaufman Assessment Digit Recall	Children with SLI (86%) have impairments in cognitive skills such as short-term phonological memory, inhibition, and processing speed.
Kuusisto (2016) [29]	22	25	4-11	Wechsler Intelligence Scale for Children, Behavior Scoring Inventory of Executive Functions (BRIEF)	Intellectual skills in children with SLI were within normal ranges for 34% of the sample; however, 64% had significantly lower results than the TD children.
Henry (2012) [30]	73	88	5-9	Battery of Working Memory Tests for Children, Nonverbal Task (ELWM), Design Fluidity, Verbal Inhibition, Motor Inhibition (VIMI), Classification Test (D-KEFS), Trail Creation Test (D-KEFS)	78% of children with SLI showed marked difficulties in various EF tasks, even when the analysis was adjusted for verbal skills.
Vugs (2015) [28]	58	58	6-10	Nonverbal intelligence test SON-R, Peabody Picture Vocabulary Test - III-NL, Reynell Developmental Language Scales	62% of SLI children had deficits in their working memory.
Schuchardt (2013) [25]	39	30	4-10	Westerman (WRT 4-5), K-ABC, DEMAT 2+, DEMAT 3+, DEMAT 4+, Weingartener (WRT2 WRT3)	86% of SLI children had deficits in their working memory.
Lukács (2015) [18]	31	31	6-11	Simple and complex tasks, simple interval: interval of digits and Corsi blocks, Complex Extension Tasks: Remembering, Listening, and Odd-one-out, N-back tasks, Stroop Tasks, Fluency tasks	69% of children with SLI showed significant alterations in their phonological working memory, inhibition, and processing speed when compared with controls.
Im-Bolter (2006) [24]	45	45	7-11	Auditory analysis skills test, Picture vocabulary test -3rd edition, Expressive vocabulary test (EVT), Formulated sentences subtest Wechsler Abbreviated Scale of Intelligence (WASI), Figure intersection test Stroop Test	77% of children with SLI had limitations in their processing capabilities, specifically in their attention inhibition skills in comparison with TD children.
Kapa (2019) [21]	58	58	3-7	Automated Working Memory Assessment (AWMA)	82% of SLI children had deficits in their EF.
Coelho (2013) [17]	28	28	3-9	Neuropsychological evaluation: Battery of Coimbra, Test of the Tower of Coimbra, Semantic and phonemic verbal fluency, Quick naming of digits, shapes, and colors	91% of SLI children had EF deficits as well as significantly worse visual and verbal memory.
Marton (2014) [22]	19	19	6-11	Culture Free Self-Esteem Inventories	46% of children with SLI are required to improve and work on their pragmatic social skills and self-esteem.
Quintero (2013) [23]	31	19	5-10	WISC-IV Reverse Modality Digits Subtest, Copy and reproduction test of a complex figure, Five-digit test, Controlled Oral Word Association Test (COWAT)	88% of the SLI group showed a significantly lower performance in EF when compared to the TD group.
Willinger (2017) [19]	61	61	3-9	WISC-IV Reverse Modality Digits Subtest, Copy and reproduction test of a complex figure, Five-digit test, Controlled Oral Word Association Test (COWAT)	76% of children with SLI show EF deficits as early as four years of age; early recognition and diagnosis are paramount for proper treatment.
Pijnacker (2016) [15]	37	25	4-8	Peabody Picture Vocabulary Quiz, Schlichting test for language comprehension, Verhoeven & Vermeer articulation task, EEG-Event Related Potentials (N400 Effect)	SLI children appear to have significantly lower levels of language and intelligence as showcased by a smaller N400 effect on EEG when compared with controls (p<0.05)
Lidstone (2012) [16]	21	21	6-10	Tower of London, Pattern Building Subtests of the British Ability Scales Test for the Reception of Grammar, Clinical assessment of the fundamentals of language	41.8% of SLI children solved the Tower of London test correctly, which was significantly lower than the TD children (p=0.01). Additionally, SLI children showed a significantly lower percentage of internalized speech than the control group.
Katsos (2011) [9]	29	58	4-9	Raven Colored Progressive Matrices, Subtest of Recall of Sentences of the battery "Assessment of Children's Language," Quantification and Informativeness, Quantification and Logical Meaning	Children with SLI have significant difficulties with pragmatics and logical meaning when compared to age-matched controls but not when compared with lower-aged language-matched controls.
Cabell (2011) [11]	59	0	6-10	Uppercase Alphabet Recognition Phonological awareness literacy assessment for preschool, Preschool Awareness of Print Words and Letters PALS-PreK Name Writing Task, PALS-PreK Rhyme Awareness Task	27% of SLI children have high emergent literacy with above mean alphabet knowledge and phonological awareness. 34% had average emergent literacy average print concepts but low alphabet knowledge. 39% had low emergent literacy with below mean alphabet knowledge, print concepts, and emergent writing.
Van Daal (2009) [14]	97	0	4-9	Articulation task of a linguistic competence test­–Taaltoets Alle Kinderen (TAK), Experimental dyspraxia battery	Decreased short-term auditory memory skills were related with deficits in language syntax skills in 63% of children with SLI and with deficits on other language skills in 28%.
Ambiado-Lillo (2023) [13]	22	22	6-10	Intelligence test for children (WISC-IV), Trail Making Test, Phono-audiological Evaluation for schoolchildren	Working memory, cognitive flexibility, executive attention, and planning skills were 100%, 50%, 77.30%, and 100%, respectively, below the mean in SLI children when compared with TD children; all associations were significant (p<0.05).
Acosta (2015) [26]	29	29	4-11	Clinical Evaluation of Language Fundamentals-3 (CELF-3), Peabody Receptive Communication, Kaufman Brief Intelligence Test (K-BIT), Illinois Test of Psycholinguistic Aptitudes (ITPA)–Auditory Association and Visual Association Subtests	43% of the children with the expressive subtype of SLI had agrammaticality, problems with verbal fluidity. and deficits in verbal and spatial working memory; furthermore, 57% of those with the expressive-receptive subtype of SLI had worse neuropsychological results overall.
Guiraud (2017) [20]	16	16	5-11	N-EEL Oral Language Assessment, Probe's nonverbal's, WISC IV, Wechsler Preschool and Primary Scale of Intelligence (WPPSI)	96% of children with SLI had a significantly reduced sensitivity for semantic inconsistencies in sentences when compared with TD children.

The available literature showcases that school-age children with SLI have significant difficulties in tasks that require EF; when compared with typical development (TD) age-matched children, SLI children present EF deficits in 62%-91% of cases as early as three to four years of age [13,14,17-21,23-25,27,28,30]. For instance, a study published in 2012 by Lidstone et al. involving 21 SLI children aged 6-10 reported a 41.8% success rate in the Tower of London test, significantly lower than controls [16]. A common deficit within EF appears to be in working memory, affecting 62%-86% of SLI children; a cross-sectional study by Ambiado-Lillo et al. showed that 100% of the studied children with SLI had working memory scores below the mean when compared to their TD peers [13,18,25,28]. Furthermore, the same study reported below mean scores in other EF such as cognitive flexibility (50%), attention (77.3%), and planning (100%) [13]. As explained before, SLI might have a direct relationship with executive dysfunction since language is important for cognitive regulation and internalized speech; certainly, some studies report significant deficits in phonological working memory, in 69%-86% of cases; short-term auditory memory, in 63% of cases; and visual/verbal memory, in 43%-91% of cases [14,16-18,20,26]. Finally, a couple of studies reported deficits in processing speed and inhibition in 69%-77% of SLI children aged 6-11 years old [18,23].

When looking into the relationship between language and EF, a study done by Cabell et al. in 59 SLI children aged four to five years, identified three sub-groups related to emergent literacy by cluster analysis as seen in Table 2; the majority of children (73%) had average or below average emergent literacy with difficulties in alphabet recognition, print concepts (working memory), emergent writing, and phonological awareness [11]. Other studies have found that SLI children have significant difficulties in understanding logical modifiers in sentences when compared with age-paired controls but not when related to language-paired controls and may also have decreased sensitivity in identifying semantic incongruencies (96% of cases) [9,10]. Finally, a cross-sectional study by Acosta et al. identified two subtypes of SLI children with significant EF deficits: an expressive subtype, 43% of cases present agrammaticality with verbal working memory and fluidity deficits; and an expressive-receptive subtype, characterized by overall neuropsychological alterations [26].

Intelligence is considered to overlap with EF in some areas and has also been a subject of research in SLI children; for instance, a study in 22 SLI children (4-11 years of age) and 25 controls by Kuusisto et al. reported that 64% of the SLI children had below average scores in the Wechsler Intelligence Scale for Children (WISC) but 34% of them were within normal ranges when compared with their TD peers [29]. Furthermore, an EEG study assessing the N400 effect reported that SLI children might have a significantly lower level of language skills and intelligence, as shown by a smaller N400 effect when compared with controls (p<0.05) [15]. Finally, some articles also reported on the effects of training in SLI children showing improvement rates that ranged from 46% to 93% in speech capabilities, pragmatic skills, internalized speech, and self-esteem [12,22].

Discussion

EF is an “umbrella” term used to encompass multiple complex cognitive abilities that serve to control other inferior cognitive skills in the pursuit of an objective or attainment of a goal; classically, they have been subdivided into three domains: working memory, task shifting, and inhibition [4]. In children, the development of EF has been linked longitudinally to multiple skills such as academic achievement, social reasoning, logic, and biological reasoning; in contrast, its deficits have been linked to different disorders like attention deficit hyperactivity disorder, autism, and depression, among others [4]. Therefore, their development is fundamental for adult life and it has been associated with different underlying neurocognitive mechanisms like inhibition, modifications of self-consciousness and reflection, an ability for abstract representations, and language development [4,5,31].

Language might influence the aforementioned underlying mechanisms for EF development in children; however, we are faced with a chicken and egg dilemma, meaning that studies have shown that EF affects language development and also that language might have a direct impact on the generation of EF [4,5]. The question is what is the directionality (EF → Language or Language → EF)? This is why we wanted to analyze the commonality of EF deficits in children with SLI and assess what subdomains are affected the most and how this might impact the progression and betterment of their language skills.

The intimate relationship between EF and language is easy to see given that the main function of language is symbolic representation by the application of a set of rules to interpret the symbols (grammar), order the symbols in a certain way for them to have meaning (syntax), and using the symbols in spoken language while modifying the pitch, intensity, and emotion (prosody) [6]. For accomplishing language production: the individual must use long-term memory, to remember and apply the rules; working memory, to keep track of what one said and what is being said by others; attention, for focusing on the message that is being received; and inhibition, to adhere to the rules or to switch from one language to another in the case of multilingualism [5,6,31]. These might be the reason why EF deficits are very common in children with SLI when compared with their TD peers; in our review, we found that studies report some type of alteration in EF (assessed by different tests, Table 2) in 62%-91% of cases [13,14,17-21,23-25,27,28,30].

The most commonly found alteration was in working memory (including phonological, auditory, and visual/verbal memory), followed by deficits in attention, processing speed, inhibition, planning, cognitive flexibility, and internalized speech [13,16,18,20,24-26,28]. Furthermore, the study by Cabell et al. managed to identify emergent literacy in SLI children by employing cluster analysis; they showed that, in general, SLI children have low emergent literacy with difficulties in symbolic recognition, writing, and phonological awareness [11]. However, most of the included studies do not discuss in detail the direction of the alterations (EF → Language or Language → EF). One study revealed that children diagnosed with SLI exhibited significant challenges in various EF tasks even when controlling for their verbal abilities, suggesting that SLI may have a negative impact on EF development [30]. Some of the included studies also pose that the restricted capacity of children with SLI to concurrently store and process verbal information may constrain their proficiency in acquiring language skills as a result of EF deficits [9,25]. However, other published studies in the literature that analyze the relationships of EF and language provide more compelling evidence for the hypothesis that language development directly affects EF as suggested by the following facts: vocabulary level in children is the differentiating factor when fulfilling an EF task, children born to deaf parents and that are native to sign language have similar performance in EF tasks as their hearing peers, deaf children have a significantly lower inhibitory control mainly due to vocabulary deficit and not due to their deafness, and multilingualism is associated with better performance in short-term and long-term auditory-verbal memory and recognition memory as shown by an investigation of 734 monolingual and 341 multilingual 9- to 10-years-old children from the Adolescent Brain Cognitive Development (ABCD) study [5,31-34].

It is important to note that in a good proportion of studies looking into EF, confounding factors are not properly controlled, and given that EF is such a broad term, encompassing multiple cognitive skills, studies often focus on a subsample of its components and analyze them through the lens of a myriad of different tests (Table 2); these leads to an increase in the heterogeneity of the available evidence and makes the establishment of a causal relationship difficult; this might be the greatest limitation of our review [4]. Additionally, as explained before, few of the included studies discuss the directionality of the intricate relationship between EF and language. However, it is important to point out that there is a considerable degree of variability in task performance among both SLI and typically developing children on measures of EF; therefore, group-level differences do not necessarily reflect the EF performance of individual members within the group since some studies show that SLI children have EF scores that fall within the performance range of their typically developing peers [11,21].

## Conclusions

The cognitive abilities of children with SLI are constrained in terms of processing capacity, inhibition, verbal and logical reasoning, attention, planning, and organization. This implies that they possess fewer cognitive resources and may not be able to utilize them efficiently. In broad terms, it may be posited that the aforementioned outcomes stem from the premise that language development might have a significant effect on EF acquisition and blossoming and that both are indispensable for cognitive growth. In the event that language acquisition does not occur in a conventional manner until the onset of formal education, it is highly probable that EF, in general, will be impacted. Consequently, these children are likely to encounter challenges in psychosocial and academic domains throughout their lifespan, relative to their typically developing peers. Therefore, comprehending the correlation between language and EF constitutes a crucial initial stride in understanding the impact of general cognitive processes on learning and functioning and in fostering optimal language acquisition.

## Appendices

Search strategy and terms used in each database

PubMed

(("Specific Language Disorder"[Mesh ]) OR (Specific Language Disorders) OR (Specific Language Impairment) OR (Specific Language Impairments) OR (Specific Language Deficiency)) AND ((Executive Function) OR (Executive Functions) OR (Executive Control) OR (Executive Controls) OR ( "Executive Function"[Mesh]))

*Scopus* 

TITLE-ABS-KEY(((Specific Language Disorder) OR (Specific Language Disorders) OR (Specific Language Impairment) OR (Specific Language Impairments) OR (Specific Language Deficiency)) AND ((Executive Function) OR (Executive Functions) OR (Executive Control) OR (Executive Controls)))

Virtual Health Library

((Specific Language Disorder) OR (Specific Language Disorder) OR (Specific Language Disorders) OR (Specific Language Disorders) OR (Specific Language Deficiency) OR (Specific Language Deficiency) OR (Impairment Language Specific)) AND ((Executive Function) OR (Executive Functions) OR (Executive Functions) OR (Executive Control) OR (Executive Controls) OR (Executive Controls) OR (Executive Controls))((Specific Language Disorder) OR (Specific Language Disorder) OR (Specific Language Disorders) OR (Specific Language Disorders) OR (Specific Language Deficiency) OR (Specific Language Deficiency) OR (Specific Language Impairment)) AND (( Executive Function) OR (Executive Function) OR (Executive Functions) OR (Executive Functions) OR (Executive Control) OR (Executive Control) OR (Executive Controls) OR (Executive Controls))((Specific Language Disorder) OR (Specific Language Disorder) OR (Specific Language Disorders) OR (Specific Language Disorders) OR (Specific Language Deficiency) OR (Specific Language Deficiency) OR (Specific Language Impairment)) AND (( Executive Function) OR (Executive Function) OR (Executive Functions) OR (Executive Functions) OR (Executive Control) OR (Executive Control) OR (Executive Controls) OR (Executive Controls))
